# Social Media Influences on Dietary Awareness in Children

**DOI:** 10.3390/healthcare12191966

**Published:** 2024-10-02

**Authors:** Victor Prybutok, Gayle Prybutok, Jesudhas Yogarajah

**Affiliations:** 1 Department of Information Technology and Decision Sciences, G. Brint Ryan College of Business, University of North Texas, Denton, TX 76203, USA; victor.prybutok@unt.edu; 2 Toulouse Graduate School, University of North Texas, Denton, TX 76201, USA; 3 Rehabilitation & Health Services Department, University of North Texas, Denton, TX 76203, USA; gayle.prybutok@unt.edu; 4 Department of Information Science, University of North Texas, Denton, TX 76203, USA

**Keywords:** childrens’ food, obesity, social media, weight management, healthy diet

## Abstract

Background: Social media platforms have become increasingly influential channels for discussing various aspects of children’s health, including dietary habits and food choices. This research explores the impact of social media on childhood dietary habits regarding the foods children consume by analyzing published findings about online videos and other social media platforms. Methods: From a pool of 9646 articles available on Google Scholar, Science Direct, Web of Science, and ProQuest, 25 were selected for inclusion in this systematic literature review after meeting the qualifying criteria. The screened papers contained clinical studies, cross-sectional studies, and editorials published in English. Results: A review of these articles revealed that individuals’ communication with friends via social media significantly enhanced their comprehension of conversations related to weight management. The results of this research inform efforts to combat poor diets and promote overall well-being among children at an earlier stage when personal interactions are likely limited. To promote the healthy growth of children, it is essential that the videos they engage with offer them appropriate guidance on maintaining a nutritious diet. Ultimately, this research provides insights into how social media creates environments conducive to the well-being of children. Conclusions: As a result, social media can serve as a valuable resource to help mitigate the prevalence of obesity in this vulnerable population.

## 1. Introduction

Children who consume nutritious diets are more likely to sustain a healthy weight and lower their chances of encountering chronic illnesses [1]. Consequently, fostering children’s early adoption of healthy eating habits becomes imperative. In this era of advancing technology, the ongoing surveillance of public discourse and the monitoring of potential shifts in sentiment or predefined categories are increasingly vital for enabling prompt responses [1,2]. The articles included examine how social media affected nutritional awareness and dietary behaviors during the COVID-19 pandemic. Another objective was to explore behavioral impacts, understand influencing factors, and examine policy implications.

In the contemporary era, photo-sharing stands out as a highly favored endeavor linked with the use of social media [3]. Consequently, both adults and younger individuals find themselves captivated by these social media platforms. The current research provides a comprehensive review aimed at addressing important alterations in snacking patterns (encompassing both sugary and savory snacks), fast-food consumption, and food ordering practices [1]. This systematic Preferred Reporting Items for Systematic Reviews and Meta-Analysis (PRISMA) review explores the literature from 2020 to 2023 on challenges related to social media’s influence on children’s weight management and dietary awareness. Social media photo-sharing features encourage users to share their daily activities, including meals and snacks, and such sharing involves a significant amount of time spent on social media [4]. Currently, there is a lack of comprehensive reviews analyzing suggested dietary changes and their link to social media. This gap in the literature lends itself to an evaluation of how social media influences children’s snacking patterns, fast-food consumption, and food ordering practices [5].

The most popular innovative trend is the promotion of healthy eating practices [3]. A quantitative aggregation of the themes found in social media advertisements created by children’s food producers and their influencers helps characterize the prevailing strategies used to increase purchases of foods by parents [6]. It is important to study how exposure to unhealthy diets on social media affects users’ eating habits and attitudes toward food, as research supports the significance of this type of exposure. Understanding children’s eating habits that result from social media influence is crucial for their long-term health [7]. The emotional expression of children on social media platforms provides valuable data for analyzing how young users emotionally respond to dietary content. This analysis allows for the assessment of both positive and negative reactions to nutritional information shared on social media [8]. Building upon this study, the current research examines how children’s nutritional needs are addressed and how social media serves as a major source of dietary information for this young demographic. The aim of this review was to investigate whether dietary awareness prevention initiatives had a clinically relevant impact on public health [9]. Specifically, the review analyzes published findings on online videos and other social media content to examine their influence on children’s food preferences and consumption patterns potentially contributing to childhood obesity trends. The current study aims to understand how food-related social media impacts children’s daily dietary choices and explores the role of parental guidance in this digital context. We explored various aspects of the relationship between social media and children’s diets with obesity.

The prevailing trend among children’s online channels incorporates visual and verbal brand elements [10]. Utilizing visuals to captivate children within food brand videos, the prevalent scenario involves a singular brand being visually represented [11]. The motivation for perusing data analysis of the posted blog content on such sites is to encapsulate the viewpoints expressed, along with contributions from parents and experts [12].

In this research two theories were applied: Social Cognitive Theory (SCT) and Objectification Theory (OT). SCT in the context of this study involves self-efficacy and observational learning of social media users [13,14]. Likewise, OT allows for the assessment of self-surveillance and includes user motivation regarding dietary change, body image, dieting body dissatisfaction, and unhealthy dieting practices.

## 2. Materials and Methods

### 2.1. Guidelines

The PRISMA statement was followed in the conduct of this review [15].

### 2.2. PICO Question

This review was designed according to PICO guidelines to determine the influence of social media on dietary awareness among children.

Population: Children exposed to social media through their parents.

Intervention: Exposure to dietary content posted on social media platforms.

Outcome 1: Increased dietary awareness and healthier eating habits among children exposed to social media dietary content.

Outcome 2: Children’s dietary choices are affected by social media advertisements that provide immediate, contactless access to dietary information.

### 2.3. Search Strategy

The protocol of this research was registered in the publicly accessible Web of Science database, as a primary database for registering systematic review protocols. This research protocol was registered to provide access to the articles identified in the search. These articles were peer-reviewed journal articles published between the years 2020 and 2023. Non-English articles, case references, duplicates, commentaries, and graphic articles were excluded to allow for empirical examination of the corpus. To conduct a methodical literature review, we completed searches in the following four databases: Google Scholar, Science Direct, Web of Science, and ProQuest. The search terms were used “children’s diet”, “children”, “overweight”, and “social media”. 

### 2.4. Study Selection

A three-step analysis process was used in this study. Numerous articles emerged during the initial step from the search engines. The first search screen initially examined the titles and abstracts of the articles, and duplicates were excluded. Subsequently, the articles were independently screened by title and abstract to identify those that broadly met the inclusion criteria. The articles were then further evaluated by examining the full manuscript. Then, health information websites, as well as articles that concentrated more on other diseases, were excluded.

The included study designs were editorials, clinical studies, cross-sectional studies, and retrospective studies.

- Articles evaluating the influence of social media on nutritional knowledge and eating habits during the COVID-19 pandemic.
- Articles involving studies performed on humans.
- Articles in English only.
- Articles with publication dates from January 2020 to December 2023.
- Studies focusing on children and adolescents aged 5–19 years old

The following exclusion criteria were applied:

Excluded study designs were case reports, bibliographic reviews, systematic reviews, meta-analyses, doctoral theses, and comparative studies.

- Articles dealing with food and nutrition that do not refer to the influence of social media on nutritional knowledge and eating behavior.
- Articles involving studies carried out on animals.
- Articles in languages other than English.
- Articles with a publication date prior to January 2020.
- Studies focusing exclusively on adults and infants (age 20 and above and below age 5)

### 2.5. Data Collection and Synthesis

**Figure 1:**
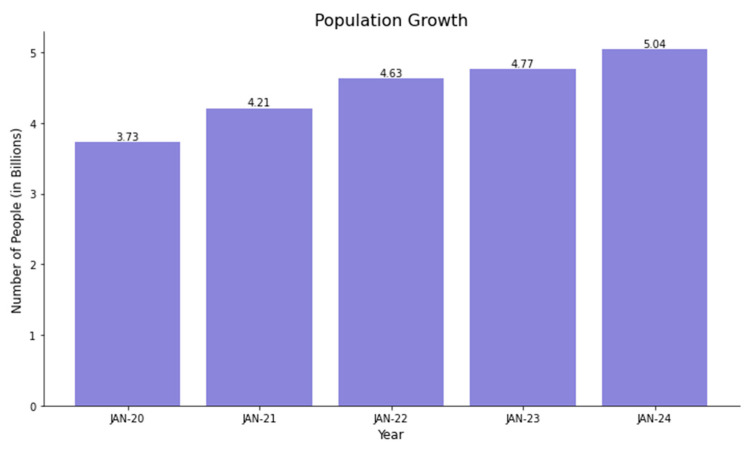
Social media trends among the population from 2020 to 2024.

This study included quantitative and qualitative research articles. The primary focus was on the usage of social media and children’s food consumption. Social media utilization increased significantly from 2020 to 2022, which is illustrated in Figure 1 [2].

### 2.6. The Quality of Included Articles

As per our objective, the articles were chosen to ensure the reliability and validity of the description of children’s diets. Another objective is to determine the risk of bias in the studies to evaluate overall strength and identify a suitable methodology that does not impact the interpretation of the results [3]. We assessed qualitative appropriateness, reliability of analysis, and data description. These high-quality articles explored how social media content shapes perceptions and knowledge about dietary choices. We aimed to explore behavior impact, understand influences, and assess policy implications [16].

Two reviewers independently evaluated the risk of bias. This evaluation was carried out following the Agency for Healthcare Research and Quality (AHRQ)-recommended approach for assessing the risk of bias in randomized controlled clinical studies, including four quality parameters, namely sequence generation, allocation concealment, incomplete outcome data, and selective reporting, and other sources of bias [17,18].

### 2.7. Data Screening and Extraction

All authors independently performed the initial screening of the title and abstract. The articles were selected providing they were considered relevant by all of the reviewers. In the event of mixed results, the authors conferred on those articles. A detailed full-text analysis was performed, and two reviewers extracted data from each study. There were no disagreements related to data screening and extraction.

### 2.8. Data Analysis

After analyzing the data, we collected data on three areas:

- Children’s food
- Social media
- Obesity

These three groups were based on the information collected from the selected articles. We decided to search for published articles from 2020 to 2023. During this period, the COVID-19 pandemic was protracted, and we saw an increase in online food and nutrition media content, which we then wanted to examine in terms of its usefulness in the food and nutrition field. We aimed to assess how useful or valuable this increased media content was for professionals or practitioners in the food and nutrition domain. The study could help identify the need for better quality control or fact-checking mechanisms in online nutrition information.

### 2.9. PRISMA Literature Review 

**Figure 2:**
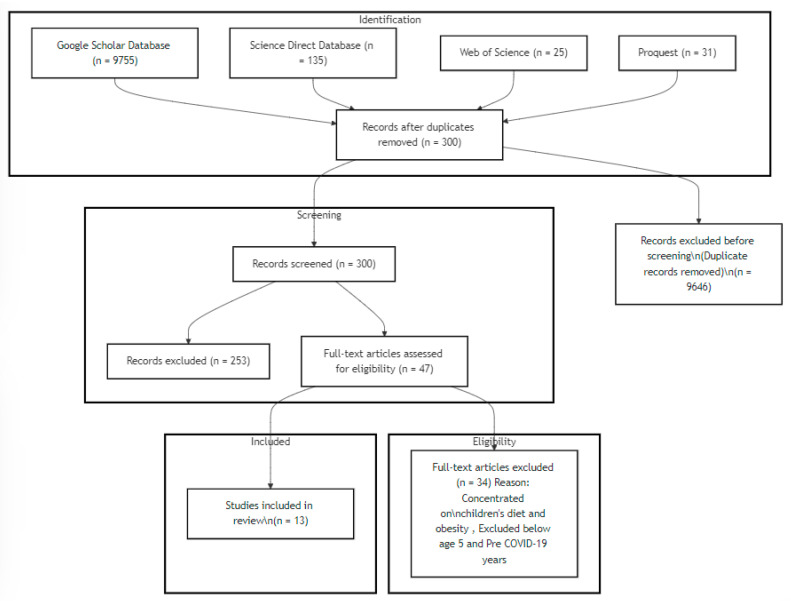
PRISMA literature review flowchart.

This systematic review began with a search across multiple databases, yielding 9946 articles. After removing duplicates and non-English publications, 300 unique citations remained. Abstract screening eliminated 47 articles, leaving 47 for full-text review. Because the focus of this review was on the impact of social media in COVID and post-COVID environments, studies that included children too young to be influenced (under 5) and studies that were conducted pre-COVID were then excluded. As a result, another 34 studies were removed. After this screening, 13 articles meeting the final criteria remained. These articles focused on food consumption and dietary awareness and did not include children younger than 5 years of age or studies that were conducted prior to the COVID-19 pandemic. The entire process, following PRISMA guidelines, is visually represented in Figure 2 of the original text, with detailed results presented in Table 1. 

## 3. Results

Table 2 shows a detailed representation of the key findings of literature reviews from 25 readily understandable articles, with the sample characteristics, research design, associates’ country of publications, and key findings presented.

### 3.1. Quality Assessment

The 25 included studies included a variety of social media platforms (Table 2). The studies were conducted in the USA, Canada, Australia, Mexico, and the UK. Topics covered included social media impacts on children’s foods, food promotion, food safety, and health promotion. All of the studies were experimental: three were RCTs and fifteen were cohort studies, and the remaining were case–control studies. Publication years ranged from 2020 to 2023, and the study durations varied from 21 days to 18 months. Participants in the studies varied in age and gender, ranging from 5 to 19 years, impacting social media as relevant to dietary awareness. Unfortunately, not all of the studies reported age data, and environmental and economic information such as food availability, food environment, and accessibility were rarely mentioned; thus, a complete characterization of the population in this meta-analysis was not possible. 

### 3.2. Assessment of Risk of Bias

The studies were graded according to high and low risk, based on selection bias, performance bias, detection bias, attrition bias, and reporting bias. The quality of individual studies was evaluated based on geographical bias, cultural bias, and sample sizes. This study focused on peer-reviewed journal articles, and inter-rater reliability was not included because it was not relevant. The expert reviewers discussed differences until agreement was reached on the terminology.

Based on the information provided in the study, some key American dietary risk habits can be inferred, especially related to children’s diets and food marketing. The study found that child influencers on YouTube frequently promote unhealthy branded food and drink products, which can influence children’s dietary preferences and behaviors. The majority of food and drinks promoted in the videos (90.3%) were unhealthy branded items, suggesting a risk of increased consumption of these products [28]. Table 2 shows a comparison between multiple countries and covers dietary habits and factors influencing them, including information about research characteristics.

### 3.3. Parents’ Motivation and Education

The use of social media involves educating parents and adolescents about healthy dietary foods and influencing healthy lifestyles through articles, videos, and food and nutrition expert interviews [29]. While many YouTubers provide valuable content promoting nutritious eating habits, there are also influencers who promote high-calorie desserts and less nutritious options through appealing posts and videos. It is important for viewers to critically evaluate the nutritional content being promoted and seek out reliable, science-based sources of dietary information [4]. Education about the value and dangers of YouTube postings is essential for enhancing parents’ comprehension of diseases such as obesity and diabetes and dental diseases. Such studies empower parents to effectively manage and prevent complications [5]. Platforms like Instagram, YouTube, and Facebook often feature content from nutritionists, while other experts offer advice and answer questions in various groups or communities [30].

COVID-19 was used in a variety of ways in food and beverage marketing on X (formerly Twitter) during our content analysis. The most common COVID-19 methods were as follows:

- Claiming that the product or service could boost the immune system, help prevent infection, or reduce the severity of symptoms of COVID-19 [30].
- Linking products or services to positive emotions associated with the pandemic. The product or service could help people feel safe, healthy, or connected during the pandemic [31].

### 3.4. Social Media Influencing Unhealthy Foods

YouTube and TikTok influence the purchase of healthy foods online through favorite food brands being advertised [1]. The findings of this study suggest that the information shared by these YouTube influencers can be inconsistent and may only sometimes be accurate [7]. In that particular study, it was found that fruit and vegetable videos did not influence young children’s intentions to engage in healthy snacks [8]. 

These types of studies can explore various aspects of food representation and discussion in media content. They may examine the correlation between nutritional content portrayed through media and engagement on social media platforms [9,10]. Such research can also reveal the contexts surrounding dietary preference discussions [8] and analyze posts related to food topics [6]. Additionally, these studies can investigate associations between daily food intake and non-communicable diseases [10,11]. Despite these policies, users may still encounter weight loss product advertisements or content that could potentially promote negative self-perception [32]. This can happen due to several factors such as inconsistent enforcement of policies, advertisers finding ways to circumvent restrictions, user-generated content that is not caught by moderation systems, and varying interpretations of what constitutes a “miraculous claim” or negative self-perception.

### 3.5. Social Media Exposure to Unhealthy Diets and Emotional Expression

In comparison with influential video creators, child influencers promoting unhealthy foods through YouTube and Instagram have significantly increased the consumption of junk food compared with influential video creators [10]. Findings on these platforms often promote high-sugar and fat foods through targeted advertisements and influencer endorsements [21,33,34]. These influencers may encourage users to consume more unhealthy dietary choices.

Additionally, the social media space for emotional expression about a broad range of foods and diets, as well as emphasizing emotional comfort, emotional eating, and other pandemic-related social challenges, results in the social isolation of children and increases feelings of loneliness [8]. It has gradually shifted to an inspirational design focusing on satisfying users’ emotional needs. Users who expressed feelings about fat stigmatization were found to be closely connected to their emotions and sought comfort from supportive communities and empathetic interactions [15]. Social media advertisements have impacted children’s eating behaviors and their adaptation to unhealthy foods as a coping mechanism for anger, anticipation, stress, anxiety, or other emotions [16]. The current research focuses on examining how three dimensions of the self (true, actual, and ideal self) influence self-expression on social networking sites and consumer behavior [16].

Another study focused on childhood diet interventions used quantitative content analysis methods [18]. In the UAE, social media has emerged as a tool to combat childhood obesity by facilitating online interactions between healthcare providers and health-conscious obese youth. A study examined this trend by analyzing content from three leading UAE newspapers and three popular social media platforms, focusing on media activities related to childhood obesity in the country. The research findings revealed that 22% of respondents expressed interest in reading stories about health concerns, suggesting a noteworthy level of engagement with health-related content. This indicates that social media and online platforms could be effective channels for disseminating information about childhood obesity prevention and promoting healthier lifestyles among UAE youth [18]. The analysis shows the UAE government’s policies and programs on childhood obesity and sharing lessons through social media of best practices on obesity prevention with UAE policymakers, parents, and other child socialization agents [35].

### 3.6. Comparison and Outcomes

Fourteen studies showed that social media had a positive impact on influencing parents and adolescents, for example, raising dietary awareness or promoting healthy diets or interventions. However, the interventions focused on uninterested populations by providing non-information-promoting strategies to promote healthy eating behaviors [36]. Most of the results show that a substantial number of these interventions either do not mention the use of theory or do not provide a comprehensive explanation of how theory guided the design of the interventions. Eleven studies demonstrated that unhealthy food is promoted through several social media platforms. The most popular innovative trend is the promotion of healthy eating practices. A quantitative aggregation of the themes found in social media advertisements posted by child food influencers helps to characterize the prevailing strategies used to increase food purchases by parents. Parents sought to address the complex relationship between child influencers and the promotion of unhealthy food choices on social media platforms [28].

## 4. Discussion

The COVID-19 pandemic created global challenges, leading to temporary measures like lockdowns to reduce social contact and limit the virus’s socioeconomic impact on individuals [3]. The food and nutrition industry, especially susceptible to concerns about children’s diets, faced dietary restrictions mandated by health officials to control the pandemic’s transmission [31]. During this period, social media communication dominated among parents for food recommendations because of the lockdown, and people preferred contactless deliveries [3]. The aim of this study was to explore the potential links between social media use, dietary behaviors, and obesity risk in children and to identify effective strategies for leveraging social media to promote healthy eating habits and nutritional education for children and families.

This study uses Social Cognitive Theory (developed in 1931) as its foundation. This theory helps explain how parents’ confidence in their abilities and their family’s mealtime habits can lead to improved knowledge and skills [37]. In addition, SCT addresses how social media supports self-efficacy, observational learning, outcome expectations, goal setting, collective efficacy, social support, and self-regulation outcomes. We address the study’s constraints, highlighting the potential for inaccuracies in self-reported data. Participants may respond based on their subjective perceptions or aim to provide satisfactory answers rather than entirely truthful ones. This tendency could lead to an overestimation of healthy eating habits in the results [38].

This study faces several potential limitations. Participants may not accurately recall their dietary habits or social media usage, leading to unreliable data due to recall bias. Additionally, self-selection bias is a concern, as the data were collected only from those who chose to engage in the study. This self-selected sample may not be representative of the general population, limiting the generalizability of the findings. Furthermore, there is a risk of confirmation bias, where researchers might unconsciously interpret the data in ways that align with their preexisting expectations or hypotheses [39,40,41,42]. Participants in the published studies noted enhancements in essential aspects linked to family mealtime practices, encompassing meal preparation, self-confidence, and the nutritional quality of meals provided to children [40].

Many studies have tried to improve people’s daily eating habits by using educational methods. These methods aim to teach people about healthy eating. However, there is a problem with a lot of these studies. They often do not talk about using any theories to guide their work, or they do not explain clearly how they used theories to create their teaching methods. This makes it hard to understand why they chose certain ways of teaching and how they thought these methods would help change eating habits. Examples of educational strategies in dietary interventions could include nutritional workshops and online classes [5,23]. Eating habits are not driven only by cognitive factors like knowledge; they are also molded by external influences surrounding the individual [8].

SCT acknowledges the concept of reciprocal determinism in behavior, where both the individual’s actions impact their environment and, in turn, the environment influences the individual [38]. SCT consists of three categories of human behavior elements: individual cognitive factors, including knowledge, expectations, and attitudes; behavioral factors, encompassing skills, practice, and self-efficacy; and environmental factors, encompassing social norms, access, and the influence of others on their surroundings [23,38]. These three factors interact synergistically to shape behavior. The factors examined were self-efficacy, practice, expectations, access, knowledge, attitude, and social norms [23,24,25]. In these investigations, which focused on promoting healthy eating, prevalent methods were nutrition workshops or classes as the evaluation of healthy eating with a focus on self-efficacy, alongside various other factors within the cognitive and environmental triads. 

During the COVID-19 pandemic, there was a noticeable increase in social media usage, with people spending more time online and consuming more health-related and home-based content. This coincided with changes in dietary habits, such as more home cooking, shifts in food accessibility, and stress-related eating. Influencers adapted by focusing more on health, immunity, and wellness topics. The pandemic also saw a rise in digital health interventions and reliance on social media for health information. Economic stress influenced food choices, impacting dietary quality and marketing strategies. When interpreting studies on social media and diet, it is important to consider the unique context of the pandemic, as pre-COVID research may not fully capture the current reality. Recommendations include conducting subgroup analyses to compare pre- and post-COVID findings and pursuing longitudinal studies to understand the long-term effects of social media on dietary habits.

### 4.1. Objectification Theory

**Figure 3:**
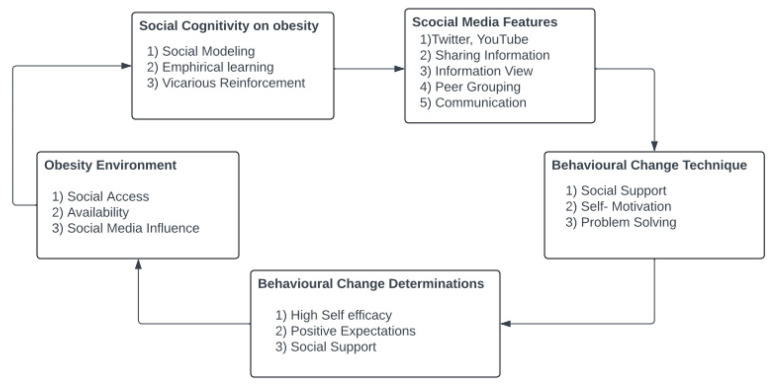
Initial and emerging factors of SCT.

OT can play a crucial role in shaping a variety of initiatives aimed at improving individual and societal well-being. Educational programs that promote positive body image can benefit from the theory by addressing the harmful effects of self-objectification, helping individuals develop a healthier relationship with their bodies [26]. Clinical interventions for eating disorders can also be informed by OT, offering deeper insights into how societal pressures and internalized objectification contribute to disordered eating behaviors and providing a foundation for future research on gendered body image and eating-related issues across various demographics, including different age groups, cultural backgrounds, socioeconomic statuses, and gender identities (Figure 3) [26]. Social media has been associated with objectification in several ways. For instance, these platforms offer individuals a space to engage in self-comparisons and self-surveillance and encounter objectifying remarks. Such experiences can result in internalized objectification, body shame, and other adverse outcomes [41]. OT can be applied to understand the internalization of body surveillance, self-objectification, and muscular dissatisfaction among men [26]. However, it is essential to recognize that objectification can affect both boys and girls [15].

### 4.2. Children’s Obesity and Health in Social Media Content

Ultra-processed foods were found in 73.6% of cases, with a higher prevalence of 79% among the subsample of child YouTubers [27]. The inclination toward healthier alternatives is manifested in several ways. The famous YouTube channel called How I Eat Junk Food & Stay Skinny says, “I stay in shape when I eat ice cream, cookies, and drinks nearly every day [31]. Here is my secret, don’t read the nutrition label”. Negative elements are more conspicuous in videos created by child YouTubers, whereas they only sporadically appear in brand videos. Child YouTubers pay relatively less attention to advocating for healthy eating habits compared to brand marketing [27]. Social media strategies that used practical advice and tools centered around the significance of providing nutritious meals instead of simply consuming more fruits and vegetables or highlighting the consequences of not doing so. Furthermore, mothers desired healthy recipes to address the time and cost challenges they encountered when striving to provide nutritious food for their families [37].

The research revealed a noteworthy positive correlation between state-level obesity rates and the average percentage of X followers who consume sugary drinks or fast-food brands [13]. Additionally, a significant, positive correlation was observed between state-level obesity rates and the mean use of hashtags related to sugary drinks and fast food on X [14].

The practical significance of school nutrition programs beyond simply providing meals to children emphasizes the need to adapt to changing dietary guidelines to provide balanced diets with essential nutrients for children [33]. Their findings are particularly noteworthy, highlighting the unexpected increase in the percentage of tweets mentioning school meals and school lunches. With the help of sentiment analysis of the collected tweets, we can assess the overall tone of healthy food [37]. This adds an insightful dimension to the study by highlighting the emotional and attitudinal aspects of online conversations about food. This method allows readers to gauge the public’s perceptions and enthusiasm toward different dietary choices, which is valuable for researchers and marketers in the food industry.

The study “Social Network Analysis of X Data” provides valuable insights into online discussions about food poverty during the COVID-19 pandemic. While it effectively analyzes tweet content and network structures, it falls short of examining the real-world impact of these digital conversations. Despite this limitation, the research offers a timely and methodologically sound exploration of how food poverty discussions unfolded on the X platform during a global crisis. This analysis reveals the strengths of the study in capturing online sentiments and network dynamics related to food poverty during the pandemic while also highlighting its primary limitation—the lack of investigation into the practical outcomes of these digital dialogues.

A substantial addition to the ongoing conversation about the impact of digital media on the eating habits of young individuals is a nutritional education program as an intervention that can effectively counteract poor diets to promote healthier dietary choices [28]. Their notable merits include their contemporary relevance, meticulous educational program, and comprehensive exploration of the wider consequences stemming from the content created by child influencers. Researchers are now including parents in their studies because they want to understand and tackle a complicated issue: child influencers on social media promoting unhealthy foods. The research focuses on child influencers (children who have large social media followings). These child influencers are often involved in promoting unhealthy food choices [28].

With children dedicating more of their leisure time to social media, young influencers’ proliferation of high-calorie food-related content is a cause for concern. Particularly noteworthy is the prevalence of chocolate as the most influential food among children on social media [11]. Additionally, the most frequently featured brands encompass McDonald’s, Kinder, and Coca-Cola, while the predominant product categories consist of chocolate, candy, soft drinks, and fruits [6,40]. Conditions/diseases such as obesity and cancer could potentially benefit the most from enhancements in clinical care through platforms like YouTube and X [18]. To promote healthy growth in children, it is essential that the videos they engage with offer appropriate guidance on maintaining a nutritious diet. Food marketing also likely affects long-term dietary consumption and may contribute to childhood obesity. 

Moreover, several researchers have sought to determine if boys and girls are differently affected by food marketing. An observational study involving preschoolers observed that girls exhibited stronger preferences for heavily promoted, branded products compared to boys [38]. However, it is important to note that this observation does not definitively establish that girls are more influenced by product advertising than boys.

### 4.3. Connection between SCT and OT

Both SCT and OT highlight the social modeling and internalization of social norms in the relationship between body shape and dietary behaviors related to dietary awareness on social media [42]. Dietary influencers and content creators, particularly those producing advertising videos on platforms like YouTube, who align with these theories should effectively promote healthy eating practices while minimizing the risk of supporting unhealthy dietary behaviors [43,44]. Educational strategies based on SCT can be used on social media to enhance users’ self-efficacy, empowering them to make informed choices about their dietary preferences [45]. Meanwhile, OT’s insights into self-surveillance and body image objectification can guide interventions to promote positive body image and healthy eating behaviors [46].

### 4.4. Limitations

It is challenging to establish the connection between the coverage of all child-based dietary samples in social media content and changes in their dietary behavior due to the observational nature of many studies [47]. The findings from some geographically based studies on certain demographic groups may not represent entire populations with different cultural norms or access to social media [48].

## 5. Conclusions

This review shows that dietary awareness using social media when personal interactions are limited, such as during the recent COVID-19 pandemic, plays a major role in enhancing dietary awareness and promoting healthy eating habits. Social media platforms can provide immediate contactless access to dietary information and guidance, allowing individuals to address dietary concerns remotely. By categorizing the level of dietary concerns, health professionals or dietary guidance systems can determine whether an individual’s issues can be addressed through social media resources or might require professional intervention. The authors believe that social media can be a valuable resource for individuals seeking information on dietary guidance, particularly those who face challenges in accessing in-person consultations. Health professionals, parents, and teachers should educate their children about media literacy and critical thinking skills to establish credible dietary information from unauthorized content on social media. These findings underscore the importance of developing comprehensive strategies that harness the positive potential of social media while mitigating its risks. By fostering digital literacy, promoting responsible social media use, and leveraging these platforms for health promotion, we can work toward creating a digital environment that supports healthy dietary behaviors and contributes to obesity prevention among young people.

## Figures and Tables

**Table 1:** Systematic review characteristics of included studies. Source: Author.

Author	Design	Social Media	Country	Key Finding
Ayoub et al., 2023 [16]	Cohort study	Facebook, Instagram, X, Reddit, Tumblr, and YouTube	Canada	Caffeinated energy drink companies are targeting young adolescents to promote their products across social media platforms to avoid obesity.
Théodore et al., 2021 [19]	Cross-sectional study	X, Facebook, and YouTube	Mexico	X allowed for real-time updates about food shortages or crises during the COVID-19 pandemic. Coca-Cola, soft drinks, and sweetened juices had the most followers on Facebook and X. Companies used diverse persuasive techniques: Promotional characters (79.1%) Incentives (65.1%) Digital techniques (78.3%)
Foubister et al., 2023 [20]	Cohort study	Facebook and YouTube	UK	Girls who spent ≥5 h/day on social media were positively associated with Body Mass Index, and it partially impacted their sleep duration, depressive symptoms, body-weight satisfaction, and well-being.
Gu et al., 2021 [21]	Randomized controlled trials	X and Instagram	USA	The study found a significant positive correlation between the social media presence of sugary drink and fast-food brands on Instagram and X and state-level obesity rates. The correlation was stronger for X compared to Instagram.
Coates et al., 2020) [10]	Case–control studies	YouTube	UK	Children displayed awareness of the persuasive intent behind influencer marketing, especially when it came to familiar YouTubers. Although they understood that influencers promote products for financial gain, they still enjoyed the content. Children believed that while influencer marketing could be effective, they felt confident in their ability to resist it. However, evidence suggests that they are still susceptible to its effect.
Meléndez-Illanes et al., 2022 [22]	Randomized controlled trials	YouTube, Instagram, and Snapchat	USA	Social media promotes harmful foods that contain saturated fats, trans fats, free sugars, and/or salt that harm child health.
Pancer et al., 2022 [9]	Randomized controlled trials	Facebook	USA	Social media events engagement and other implications for content developers, advertisers, consumer health advocates, and policymakers.
Sacks and Looi, 2020 [23]	Case–control Studies	Facebook, YouTube, WhatsApp, WeChat, Instagram, QQ, QZone, Douyin/TikTok; SinaWeibo, Reddit, X, Douban, Snapchat, LinkedIn, and Pinterest	Australia	Social media platforms have policies restricting the advertising of unhealthy diets/lifestyles, such as those involving alcohol, tobacco, gambling, and weight loss. and are contributing to efforts to improve population diets.
Tsai et al., 2022 [6]	Randomized controlled trials	X	USA	The majority of tweets (n = 682, 78%) promoted foods and beverages during the COVID-19 pandemic. This became the root cause of nutrition-related diseases such as obesity and diabetes.
Van der Bend et al., 2022 [24]	Cohort study	Instagram, Snapchat and YouTube	Australia	Adolescents’ social media food promotions exposed unhealthy foods through advertisements integrated into various forms of entertainment that they enjoy.
Whitesell and Fitch, 2022 [25]	Case–control studies	TikTok, Snapchat, X, And Instagram	USA	TikTok can be an engaging way to reach adolescents’ desired health information on physical fitness and diet.
Winzer et al., 2022 [26]	Cohort study	TikTok, YouTube, and Instagram	UK	Nutritionally poor food content targeted at children.
Zhang et al., 2021 [27]	Cohort study	Facebook	USA	Facebook influences cooking posts among low-income caregivers.

**Table 2:** Comparison of dietary factors and research characteristics across selected countries.

Country	Bias
USA	Dietary habits: High consumption of processed foods, sugar-sweetened beverages, and fast food Geographical bias: Urban vs. rural differences and food deserts in some areas Cultural bias: Diverse ethnic backgrounds influencing diet Sample sizes: Large national surveys (e.g., NHANES) with thousands of participants
Canada	Dietary habits: Like in the USA but with some regional variations Geographical bias: Urban vs. rural and north vs. south differences Cultural bias: Multicultural influences, especially in urban areas Sample sizes: Medium to large national surveys (e.g., the Canadian Community Health Survey)
UK	Dietary habits: Concerns about high sugar and fat intake but efforts to improve school meals Geographical bias: Some north–south divide in dietary patterns Cultural bias: Multicultural influences in urban areas Sample sizes: Large national surveys (e.g., the National Diet and Nutrition Survey) with thousands of participants
Australia	Dietary habits: Similar to other Western countries, with high intake of discretionary foods Geographical bias: Urban vs. rural differences and remote areas may have limited food access Cultural bias: Increasing multicultural influences, especially in cities Sample sizes: Medium to large national surveys (e.g., the Australian Health Survey)
Mexico	Dietary habits: Coca-Cola and soft drinks, sweetened juices, and energy beverages were among the products with the greatest number of followers on social media platforms, and free sugars made up 13% of energy intake in Mexican children’s diets Geographical bias: Digital marketing of food and beverage products in Mexico Cultural bias: The food and beverage categories analyzed were based on products commonly promoted to children Sample sizes: A total of 46 food/beverage products from Mexican websites; 41 Facebook accounts; 40 X accounts; and 33 YouTube accounts were analyzed. A total of 129 digital marketing platforms were studied.

## Data Availability

Not applicable.
